# Morphometric Analysis of the Sphenoid Sinus Ostium in Computed Tomography: Implications for the Endonasal Transsphenoidal Approach in Pituitary Neuroendocrine Tumor Surgery

**DOI:** 10.7759/cureus.96377

**Published:** 2025-11-08

**Authors:** Jonathan Ortiz-Rafael, Isaac J Palacios-Ortiz, Angel R Martinez-Ponce de Leon, Arturo Sotomayor-Gonzalez

**Affiliations:** 1 Department of Neurological Surgery, Dr. José Eleuterio González University Hospital, Autonomous University of Nuevo León, Monterrey, MEX; 2 Department of Neurological Surgery, Monterrey Regional Hospital, Institute for Social Security and Services for State Workers, Monterrey, MEX

**Keywords:** endonasal endoscopic approach, pituitary neuroendocrine tumor, preoperative imaging, skull base surgery, sphenoid sinus ostium

## Abstract

The sphenoid sinus ostium represents the main anatomical landmark for endoscopic endonasal approaches to the sellar region, which serves as a safe entry corridor into the sphenoid sinus. Characterization of this sinus anatomy is essential for preoperative planning in pituitary neuroendocrine tumor surgery. This study aimed to analyze the morphometric features of the sphenoid sinus ostium to optimize surgical preparation.

A retrospective study was conducted on computed tomography scans of 30 control individuals bilaterally (60 sides) and 30 pituitary neuroendocrine tumor patients bilaterally (60 sides). Measurements included distances from the sphenoid sinus ostium to the anterior nasal spine, sphenoid sinus floor, sphenoid sinus ceiling, and anterior sellar wall. Angles between the nasal spine and the ostium to the sellar tuberculum and sellar floor were measured.

In the control group, the mean sellar and tubercular angles were 155.5° (SD ± 12.29) and 168.5° (SD ± 9.64) respectively. Longer distances to the sphenoid sinus ceiling correlated with reduced tubercular angles and increased sellar angles, while greater anterior sellar wall distance correlated with larger sellar angles. Pituitary neuroendocrine tumor group displayed a sellar angle of 132.0° (SD ± 15.49) and tubercular angle of 158.7° (SD ± 13.20), both statistically reduced compared to the control group.

Sellar and tubercular angles may predict surgical endoscopic trajectory. When these angles are reduced, extensive removal of the posterior ethmoid cells and sphenoid rostrum may be required to adequately expose the tubercular recess and sellar floor, respectively. Preoperative analysis of sphenoid sinus ostium morphometry for surgical planning may guide the extent of bone removal needed, enhancing safety in pituitary neuroendocrine tumor surgery.

## Introduction

The sphenoid sinus (SS) is a central anatomical region in the posterior paranasal sinus system at the base of the skull. The SS lies in close proximity to critical neurovascular structures and anatomical regions such as the pituitary gland, cavernous sinus and its contents (carotid artery, cranial nerves III-VI), central surface of the brainstem, among others, making it a significant structure in sellar and parasellar surgery [1]. At the anterior wall of the SS lies the sphenoid sinus ostium (SSO), an aperture that typically communicates with the nasal cavity draining into the sphenoethmoidal recess [2].

The SSO represents the main anatomical landmark for access to the SS in the endoscopic endonasal approach (EEA), as it is the safest place for entrance without injuring contiguous structures and allows access to the sellar and parasellar regions [3]. The SSO also serves as a reliable radiological landmark that can be located in preoperative imaging, particularly in high-resolution paranasal sinus computed tomography.

The SS anatomy can be greatly distorted by the presence of pituitary masses, making the exposition of the sellar and parasellar regions challenging. Understanding of the anatomy of the SSO and its neighboring structures is crucial in the planning and execution of EEAs, and presurgical analysis using imaging studies can be helpful in predicting the operative strategy and the extent of bone removal required for adequate sellar exposure.

Despite the surgical relevance of the SSO, few studies have analyzed its morphometric characteristics to enhance surgical planning. The aim of this study is to emphasize the role of the SSO anatomical characteristics and relationships that can optimize surgical safety, improving access routes planning to the sellar region.

A preview of this work was sent to be presented as a meeting abstract at the 2026 NASBS Annual Scientific Meeting on March 4-6, 2026 in San Diego, CA.

## Materials and methods

Study design

Retrospective radiological review of paranasal sinus thin-slice (<1 mm) computed tomography scans in bone window obtained from a tertiary center picture archiving and communication system (PACS) database in healthy subjects and pituitary neuroendocrine tumor (PitNET) patients with sellar and postsellar SS pneumatization.

Study population and sample size

A control group was formed by 30 patients older than 14 years where computed tomographies in the bone window were analyzed bilaterally (60 sides). Patients with conchal SS, skull base trauma, with sellar or paranasal tumors that can distort the local anatomy, or with prior surgical approaches to the sellar or paranasal sinus regions were excluded. The control group was compared with a PitNET group consisting of thin-slice computed tomography in the bone window of 30 patients with pituitary macroadenoma (>10mm in diameter) analyzed bilaterally (60 sides), patients with previous surgical approaches to the sellar or parasellar sinus region, conchal SS, and with adenoma invasion to the sphenoid caused by sellar floor destruction were excluded. Age and sex were considered for analysis in both groups.

Study measurements

Anatomical landmarks included in relationship to the SSO were the anterior nasal spine (ANS), sphenoid sinus floor at level of SS anterior wall (SSF), sphenoid sinus ceiling at level of the anterior wall of the SS (SSC) the anterior wall of the sella (AS), sellar floor (SF), the bony palate (BP), and the tuberculum sellae (TS).

**Figure 1:**
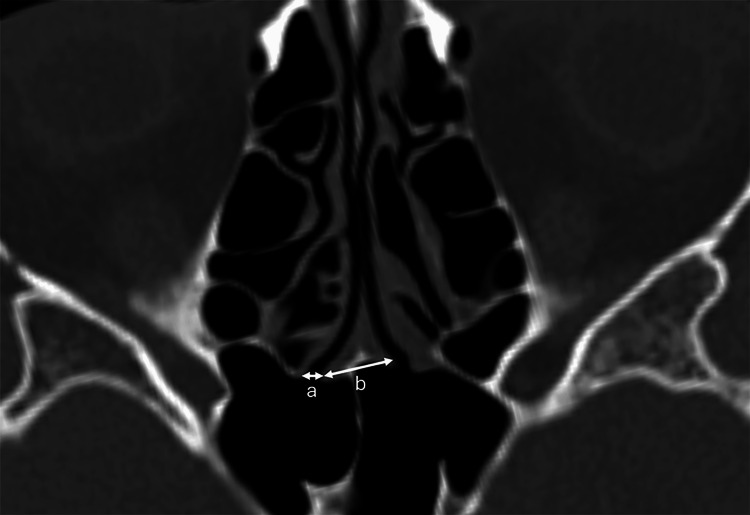
Axial plane lengths a. sphenoid sinus ostium diameter (SSO diameter). b. inter-sphenoid sinus ostium distance (SSO distance)

**Figure 2:**
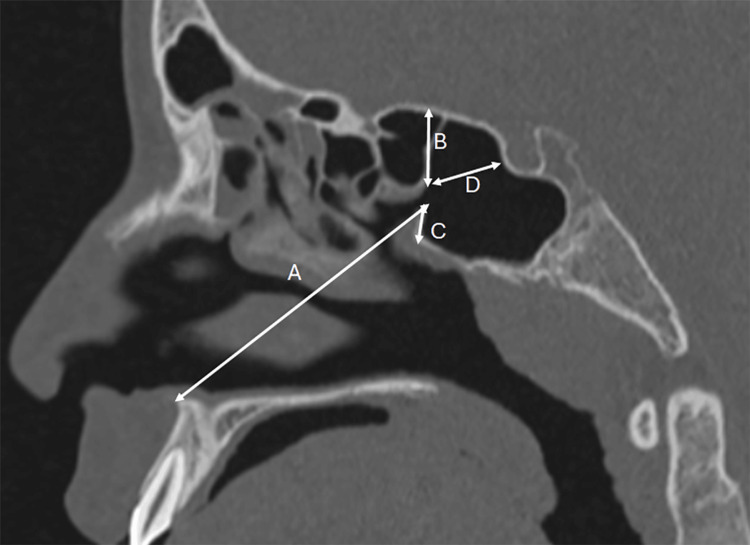
Sagittal plane lengths A. sphenoid sinus ostium-anterior nasal spine (SSO-ANS). B. sphenoid sinus ostium-sphenois sinus ceiling (SSO-SSC). C. sphenoid sinus ostium-sphenoid sinus floor (SSO-SSF). D. sphenoid sinus ostium-anterior wall of the sella (SSO-AS)

Measurements were taken in both axial and sagittal planes using multiplanar reconstructions. In the axial plane, the diameter of the SSO was measured from its medial to lateral margin, and the distance between the medial borders of both sphenoid ostia (inter-SSO distance) was recorded (Figure 1). In the sagittal plane, four distance measurements were made: from the lower edge of the SSO to the ANS (SSO-ANS); from the lower edge of the SSO to the sphenoid sinus floor (SSO-SSF); from the upper edge of the SSO to the anterior wall of the sella (SSO-AS); and the upper edge of the SSO to the sphenoid sinus ceiling (SSO-SSC) (Figure 2).

**Figure 3:**
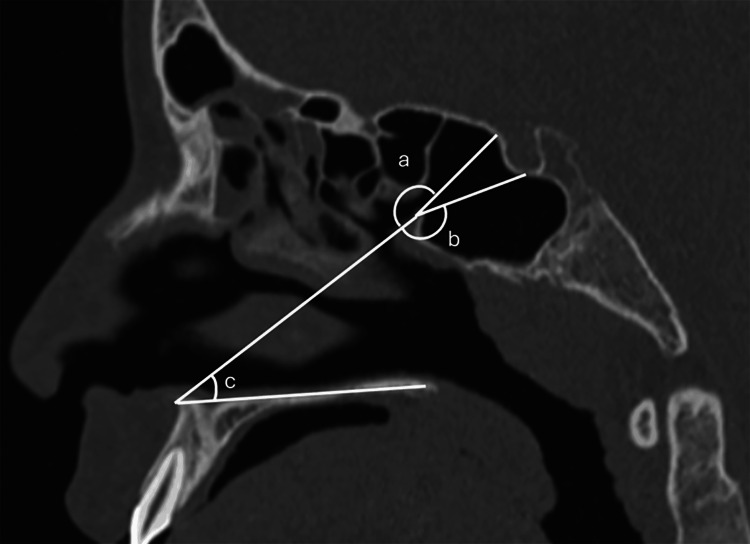
Sagittal plane angles a. Nasal angle (NA). b. Tubercular angle (TA). c. Sellar angle (SA).

Additionally, three angles were measured in the sagittal plane. The nasal angle (NA) was defined as the angle formed between the posterior edge of the bony palate and the inferior margin of the SSO, using the ANS as the vertex. The sellar angle (SA) was formed between lines drawn from the ANS to the SF, with the vertex at the inferior margin of the SSO, and the tubercular angle (TA) was measured between lines drawn from the ANS to the TS, also using the inferior SSO margin as the vertex (Figure 3).

Statical analysis

Statistical descriptive analysis was performed in statistical software SPSS 29.0 (IBM Corp., Armonk, NY, USA) to analyze these findings. Each side was treated as independent observations for statistical study. Normality was assessed using the Kolmogorov-Smirnov test. Pearson/Spearman tests were performed to correlate the lengths and angles in the control group depending on the variable normality. Statistical comparison between the control group and the PitNET group was carried out using an unpaired Mann-Whitney U test. A p value <0.05 was considered as statistically significant.

Ethics statement

This study was conducted in accordance with the principles of the Declaration of Helsinki. Patients were informed that imaging data might be used for publication in scientific literature. The study used anonymized imaging data, and no identifiable patient information was reported.

## Results

In the control group, 15 individuals were male (50%) and 15 were female (50%). Mean age was 49 years old (SD ± 16.56) ranging from 14 to 73 years. In the axial plane lengths, mean SSO diameter was 2.39 mm (SD ± 0.92), mean inter-SSO distance was 5.64 mm (SD ± 1.95). In the sagittal plane linear distances, SSO-ANS mean distance was 54.85 mm (SD ± 8.14), mean SSO-SSC distance was 8.96 mm (SD ± 3.23), mean SSO-SSF distance was 6.79 mm (SD ± 2.29), SSO-AS mean distance resulted in 13.29 mm (SD ± 2.89). The sagittal plane mean angles were: tubercular angle 168.48° (SD ± 9.64), sellar angle 155.49° (SD ± 12.29), nasal angle 37.00° (SD ± 3.23). Longer SSO-SSC lengths corresponded to reduced TA (r= -0.38) (p=0.03), and greater SA (r=0.70) (p=0.00), larger NA correlated to reduced SA (r=-0.40) (p=0.01). Larger SSO-AS lengths correlated to larger SA (r=0.46) (p=0.00), SSO-AS length did not correlate to TA (r=0.06) (p=0.63). SSO-SSF length did not correlate to the TA (r=0.26) (p=0.04) nor the SA (r=0.14) (p=0.25) (Table 1).

**Table 1:** Correlations Between Anatomical Measurements and Angles

	Correlation (r)	p-value	Interpretation
Sphenoid sinus ostium-sphenoid sinus ceiling (SSO–SSC) length vs. Tubercular angle (TA)	-0.38	0.03*	Moderate inverse correlation
Sphenoid sinus ostium-sphenoid sinus ceiling (SSO–SSC) length vs. Sellar angle (SA)	0.70	< .01*	Strong positive correlation
Nasal angle (NA) vs. Sellar angle (SA)	-0.40	< .01*	Moderate inverse correlation
Sphenoid sinus ostium-anterior sellar wall (SSO–AS) length vs. Sellar angle (SA)	0.46	< .01*	Moderate positive correlation
Sphenoid sinus ostium-anterior sellar wall (SSO–AS) length vs. Tubercular angle (TA)	0.06	0.63	No significant correlation
Sphenoid sinus ostium-sphenoid sinus floor (SSO–SSF) length vs. Tubercular angle (TA)	0.26	0.04*	Weak positive correlation
Sphenoid sinus ostium-sphenoid sinus floor (SSO–SSF) length vs. Sellar angle (SA)	0.14	0.25	No significant correlation

**Figure 4:**
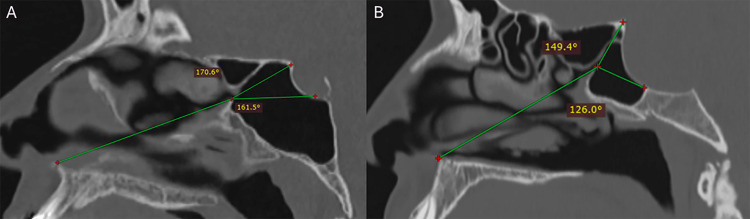
Sellar anatomy remodeling caused by pituitary neuroendocrine tumor growth results in reduction of the tubercular and sellar angles A. control individual tomography. B. pituitary neuroendocrine tumor patient tomography

The PitNET group consisted of 17 male patients (56.66%) and 13 female patients (43.33%). Mean age was 52 years old (SD ± 18.25) ranging from 19 to 75 years. Axial plane measurements included: SSO diameter with a mean of 2.1 mm (SD ± 0.69), mean inter-SSO distance was 6.19 mm (SD ± 2.20). Sagittal plane measurements included: SSO-ANS mean distance 55.43 mm (SD ± 12.88), mean SSO-SSC distance was 8.20 mm (SD ± 2.46), mean SSO-SSF distance was 6.73 mm (SD ± 2.08), SSO-AS mean distance was 9.78 mm (SD ± 3.04). The sagittal plane mean angles measurements included: tubercular angle 158.71° (SD ± 13.20), sellar angle 131.99°(SD ± 15.49), nasal angle 37.18°(SD ± 9.97). Morphometric measurements between the control and PitNET groups' means were compared. No significant differences were observed for SSO diameter (p=0.101), SSO-SSC distance (p=0.159), SSO-SSF distance (p=0.811), SSO-ANS distance (p=0.069), or the NA (p=0.781). In contrast, significant differences were detected in SSO-AS distance (p<0.001), TA (p<0.001), and SA (p<0.001). Overall, a reduction in angular sellar measurements was observed (TA and SA) and in the spatial relationship of the SSO to the sella, while anatomical structures further to the sella remain comparable to controls (Figure 4). Mean measurements in the control and PitNET group are shown in Table 2.

**Table 2:** Mean measurements comparison between groups

	Control Group	PitNET Group	p-value
SSO diameter	2.39mm ± 0.92	2.10mm ± 0.69	0.10
Inter-SSO distance	5.64mm ± 1.95	6.19mm ±2.20	0.33
SSO–ANS distance	54.85mm ± 8.14	55.43mm ± 12.88	0.07*
SSO–SSC distance	8.96mm ± 3.23	8.20mm ± 2.46	0.16
SSO–SSF distance	6.79mm ± 2.29	6.73mm ±2.08	0.81
SSO–AS distance	13.29mm ± 2.89	9.78mm ± 3.04	< .01*
Tubercular angle (TA)	168.48° ± 9.64	158.71° ± 13.20	< .01*
Sellar angle (SA)	155.49° ± 12.29	131.99° ± 15.49	< .01*
Nasal angle (NA)	37.00° ± 3.23	37.18° ± 9.97	0.78

## Discussion

The SS serves as the primary anatomical corridor for accessing the skull base during endoscopic endonasal surgery. The SSO represents the safest entry point into the sinus as it denotes a natural passage point communicating the nasal cavity and the SS, making it a key anatomical landmark to guide the surgical approach [4,5]. The location of the SSO in the anterior wall of the SS can be affected by the process of developmental pneumatization and location of adjacent bony structures [3]; therefore, presurgical location of the SSO using imaging studies is critical in the EEA planning.

The present study provides morphometric characterization of the SSO and its relationships to the sellar region. Prior series have established variability in SSO dimensions: SSO diameter and inter-SSO distance found in this study were slightly lower than that described by Jaworek-Troc et al. (0.31±0.09 and 0.63±0.28 respectively) [6]. In a cadaveric study, Campero et al. described great variability in the distance between the internal margin of the ostia and the midline, where in some skulls the SSO is located relatively far from the midline, and in some skulls the SSOs are practically adjacent to one another, consistent with the spectrum observed in the axial measurements we reported [7]. Hyun-Ung et al. reported in a cadaveric study the distance from the limen nasi to the SSO, finding similar results to this study SSO-ANS distance (56.5±3.2mm) [2]. In an imaging study, Hwang et al. described variations in the SSO-SF, SSO-SC distances comparing groups of patients with presence and absence of Onodi cells, in which the Onodi group presented lower values in both distances; SSO-AS distances of the non-Onodi group and Onodi group were similar to the one presented in this study (12.8 ± 1.1 and 13.8 ± 0.8 respectively) [8].

Beyond linear distances, various studies have explored angular parameters used in the aid of anatomical location and surgical difficulties prediction for the endoscopic approaches to the skull base. Davis et al. reported the SSO location to be 30° and 7cm relative to the horizontal plane of the anterior nasal spine [9]. Zhao et al. described the angle from the limen nasi, the SSO and the horizontal line to be 37.5° in healthy subjects in a tomographic study [4]. Tomographic angle measurements have been used for EEA surgical planning for sites other than the SSO. Kołodziejczyk et al. proposed an angle between the nasal floor and the plane tangent to the posterior table of the frontal sinus above the most posterior aspect of the anterior buttress that can help predict difficult access to the frontal sinus [10].

**Figure 5:**
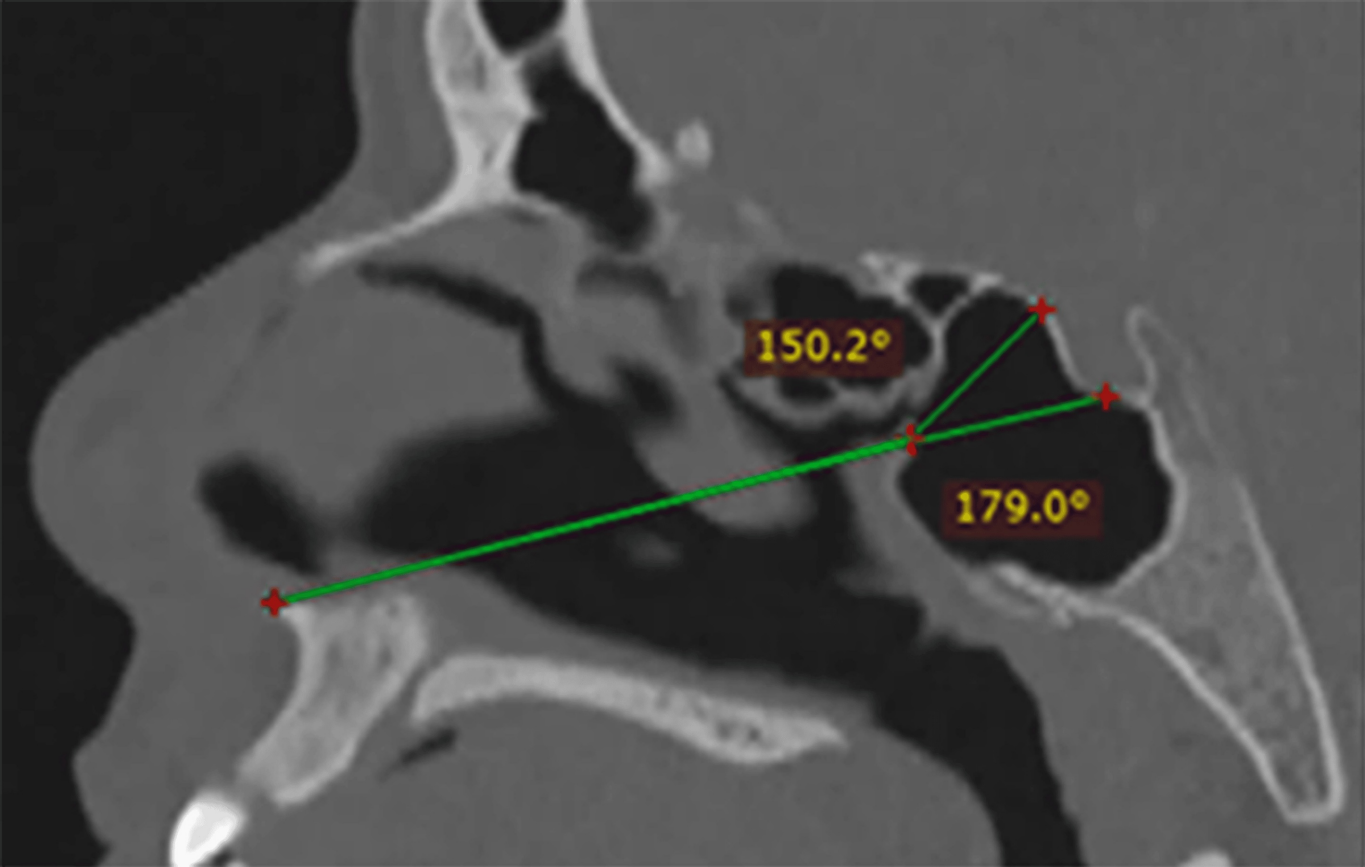
High sella Reduced Tubercular Angle and straight Sellar Angle. Might require extensive posterior ethmoidal cell resection

**Figure 6:**
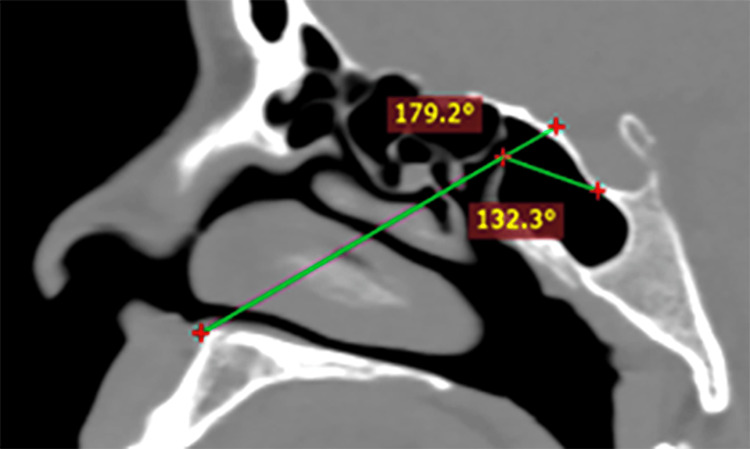
Low sella Near straight Tubercular Angle and reduced Sellar Angle. Might require extensive sphenoidal rostrum drilling

**Figure 7:**
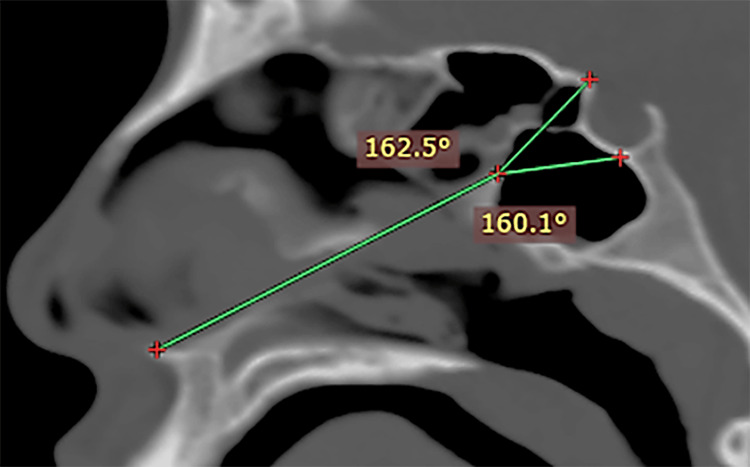
Middle sella No clear straight Tubercular Angle or Sellar Angle can be identified

We propose the use of the tubercular angle and the sellar angle in relation to the SSO as means of presurgical EEA planning to the sellar and parasellar regions. The ANS was used as the initial reference point, as it is a distal bony structure with more consistent anatomical reliability compared to the surrounding soft tissue landmarks. The TS and the SF were designated as terminal points, as their exposure is necessary for proper visualization during sellar surgery, and is critical to achieving maximal tumor resection. The introduction of the endoscope lens follows a similar trajectory to that from the ANS to the SSO, therefore, structures approaching a straight 180° angle from this perspective will be more easily identified in a primary view, in contrast, when these structures deviate further from the straight 180° angle, their identification becomes more challenging, often requiring more extensive drilling of the adjacent bone to achieve adequate exposure. Following this premise, we propose the classification of sellar height based on its relationship to the SSO in three types (Table 3): 1) High sella- with near straight SA and reduced TA (Figure 5), 2) Low sella- with near straight TA and reduced SA (Figure 6), and 3) Middle sella- with no clear straight SA or TA (both <165°) (Figure 7). High sellae and reduced TA will require more extensive removal of the posterior ethmoid to expose the TS, whereas low sellae and reduced SA will need extended drilling of the sphenoidal rostrum to achieve adequate exposure of the SF. The 165° threshold was selected as a deviation greater than 15° from a straight (180°) trajectory would limit endoscopic visualization of the sellar structures, thereby requiring additional bone removal of the posterior ethmoid or sphenoid rostrum to achieve adequate exposure. Angles greater than 165° would get closer to the straight (180°) trajectory, allowing direct endoscope visualization and requiring less bone removal.

**Table 3:** Sellar height classification based on sellar and tubercular angles

Sellar height	Characteristics
High Sella	Straight sellar angle (>165°) and reduced tubercular angle (<165°)
Middle Sella	Reduced sellar and tubercular angle (both <165°)
Low Sella	Reduced sellar angle (<165°) and straight tubercular angle (>165°)

**Figure 8:**
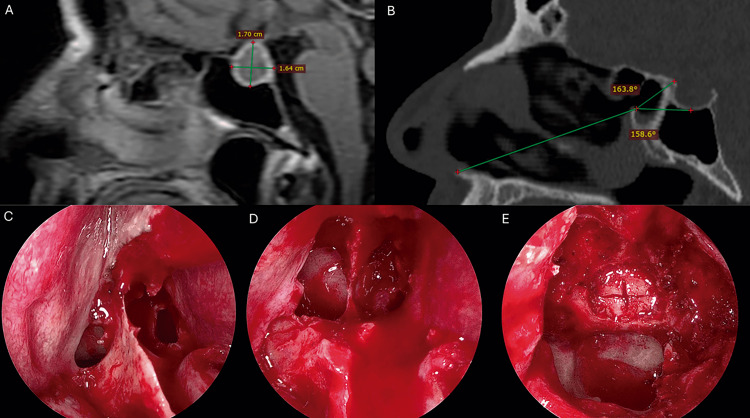
Non-functioning pituitary neuroendocrine tumor endoscopic endonasal transsphenoidal resection in a 72-year-old male A. Sagittal contrast enhanced magnetic resonance image displaying pituitary macroadenoma. B. Sagittal computed tomography exhibiting reduction of the Sellar and Tubercular angles. C. Sphenoidal rostrum exposition. D. Initial sphenoidotomy. E. Extended sphenoidotomy: posterior ethmoidectomy to expose tubercular recess, and rostrum removal to expose the sellar floor and clival recess, sellar floor drilling and cross-shaped aperture of the sellar dura is shown.

PitNET growth tends to promote progressive remodeling of the sella turcica, which in turn reduces the pneumatized volume of the SS. We observed a generalized decrease in the measurements of the sagittal SS angles (SA and TA), as well as in the SSO-AS distance. From a surgical perspective, preoperative assessment of these morphometric parameters may provide a reliable value for estimating the extent of tumor-induced remodeling, and consequently, such measurements can be helpful in predicting the degree of bone resection (posterior ethmoidal cells and sphenoid rostrum) required to achieve adequate exposure of both the tubercular recess and the sellar floor. Careful evaluation of these anatomical modifications may be useful in optimizing the endoscopic surgical corridor, potentially improving visualization and facilitating complete PitNET resection. This angle-based imaging surgical planning may be particularly beneficial for young neurosurgeons, skull base surgery fellows, and neurosurgery residents. Figure 8 illustrates a case of a 72-year-old man with a non-functioning PitNET that underwent endoscopic endonasal transsphenoidal resection surgery, exhibiting reduction of the SA and TA. Davis et al. and Zhao et al. described anterior nasal spine-based approach angles for surgical guidance but only on healthy subjects and did not analyze tumoral remodeling effects [4,9]. Our study extends these findings by showing that TA and SA are significantly reduced in PitNET patients, suggesting that mass-caused sellar remodeling alters the endoscopic view required to expose the tubercular recess and the sellar floor, and may necessitate more extensive posterior ethmoid or rostrum removal.

Limitations of this retrospective study include the use of computed tomography from a single center, which may limit generalizability. Computed tomography static measurements were analyzed, without magnetic resonance correlation. In addition, patients with invasive tumors or markedly distorted anatomy were not included; therefore, the observed reduction in the TA and SA for predicting posterior ethmoid or rostrum removal may not be applicable in such cases.

## Conclusions

Sphenoid sinus ostium represents the main anatomical landmark for access to the sphenoid sinus, as it is a natural corridor that communicates the nasal cavity and the sellar and parasellar regions. Presurgical planning based on the sphenoid sinus ostium presented in this study can provide valuable guidance for surgical planning, particularly the angles of the tuberculum and sellar floor; when these angles approach 180° in the sagittal plane, the corresponding structures are expected to be easily identifiable during transsphenoidal surgery. Conversely, a reduced tubercular angle may require more extensive drilling of the posterior ethmoidal cells, whereas a decreased sellar angle may involve increased drilling of the sphenoidal rostrum to achieve adequate surgical exposure. These results describe morphometric associations rather than causal relationships, and the clinical implications should be interpreted within the descriptive nature of the study.

Pituitary endocrine tumors notably distort the sellar anatomy, making its endoscopic approach potentially difficult, using the tubercular and sellar angles as a guide in presurgical planning for pituitary adenoma resection, especially in young neurosurgeons, skull base surgery fellows, and neurosurgery residents, can be beneficial for predicting the extent of bone drilling required for exposure of sellar and parasellar regions, enhancing safe total tumor resection.
